# Comparing Remifentanil Versus Propofol Effect on Pain and Homodynamic Change of Patients Undergoing Phacoemulsification With Topical Anesthesia

**DOI:** 10.5812/ircmj.2316

**Published:** 2013-05-05

**Authors:** Dawood Aghadoost, Mohammad Reza Fazel, Esmaiel Fakharian

**Affiliations:** 1 Trauma Research Center, Kashan University of Medical Sciences, Kashan, IR Iran

**Keywords:** Remifentanil, Propofol, Anesthesia

## Abstract

**Introduction:**

The purpose of this study was to compare remifentanil versus propofol effect on pain and homodynamic in patients undergoing phacoemulsification with topical anesthesia.

**Materials and Methods:**

A double blind clinical trial was conducted to research following the approval of the ethical committee research of the university. One hundred volunteer subjects were randomly assigned into two equal groups (n = 50). The subjects in the propofol group received 3mg/kg/hr while the patients in the remifentanil drug received 3 µg/kg/hr of this medication. Phaco time, blood pressure and heart rate before and after surgery, respiratory depression (O2 sat < 90%) and vomiting, pain scores, ophthalmologist satisfaction and demographic data were recorded.

**Results:**

The results of analysis showed that there were no significant differences between the age, sex, and duration of operation of the two treatment groups. Systolic, diastolic blood pressure and heart rate were significantly lower in the propofol group .The propofol group complained of pain than the remifentanil group (P = 0.001) while the surgeon satisfaction was higher for the remifentanil condition (P = 0.01). No significant differences were found between the two groups with respect to respiratory depression .No patient suffered from nausea and vomiting.

**Conclusions:**

The results of this study indicated that using appropriate dose of remifentanil instead of propofol results in less pain, more stable homodynamic condition, and satisfaction of surgeon without no respiratory depression or perioperative nausea and vomiting.

## 1. Background

Cataract is one of the most prevalent surgical operations performed in old ages. Approximately 1500000 cases of cataract surgery are performed annually in the United States (1).1 General anesthesia, retrobulbar and prebulbar blocks are associated with many complications (2-5). Recently, topical anesthesia is widely accepted in cataract procedures, and has become the first choice in most cases of planned routine cataract surgery (6). Pain experience in topical anesthesia can lead to complications.

Thus, applying analgesic medicine to alleviate pain is necessary. Narcotic drugs, propofol, and benzodiazepines are the drug of choice and have been employed to some extent and have led to the reduction of anxiety and pain in patients (7-9). More research is needed to identify drugs that have the most benefits and least side effects. Remifentanil is a selective narcotic agonist with analgesic power and chemically it belongs to fentanyl group. It has estery structure and high metabolic rate and has liver and out of liver elimination path. In addition, kidney and liver failure has no effect on medicine metabolism of this drug (10). Propofol is one of the medicine belonging to the alkylphenols derivative that is insoluble in water in room temperature and soluble in fat and has high metabolism rate. This study was designed to evaluate the effectiveness of this analgesic substance in pain relief, blood pressure and heart rate changes, surgeon satisfaction, nausea and vomiting during the operation in compare to remifentanil in phacoemulsification surgery using topical anesthesia.

## 2. Materials and Methods

This study was conducted by using a double blind clinical trial research design following the approval of ethical research committee of Kashan University of Medical Science (KAUMS) on 100 patients scheduled for phacoemulsification surgery from December of 2009- April 2010 in Matini Eye Center Hospital of Kashan. Before participating, patients gave their written informed consent. The patients were randomly assigned into two equal groups (n = 50). Following the admission in the operation room, interavenous line was set then heart rate, blood pressure and arterial oxygen saturation was assessed. The exclusion criterion were conditions such as dementia, severe loss of hearing or vision sufficient enough to cause cooperation failure, mental retardation, uveitis, history of eye trauma and glaucoma. For topical anesthesia tetracaine drop was used four consecutive times within 5 minute time interval. The subjects in the propofol group received 3mg/kg/hr while the patients in the remifentanil group received 3 µg/kg/hr of medication.

If patient complained of pain, the amount of medication was increased by 1mg/kg/hr in propofol and 1 µg/kg/hr in remifentanil group and when the respiratory depression observed, the amount of drugs was reduced. Following the start of the surgery procedure and every 5 minutes, blood pressure and heart rate was measured. At least 3 minute after the start of the medication administration phacoemulsification and foldable implantation was done in routine procedures. At the end operation, the data including intensity of pain during the operation was assessed by using visual analog scale (VAS- scoring range 0 to 10), surgeon satisfaction (rating excellent to bad), frequencies of respiratory depression (O2 sat < 90%), and nausea and vomiting was recorded. These data in addition to the demographic characteristics of the patients were analyzed using SPSS software. Independent T-test used for comparing mean age, phaco time and mean pain between groups < t-student used for comparing change in blood pressure and heart rate before and after beginning of surgery and Chi squared test were employed to comparing sex, distribution of the patients according to the pain rating scale during the operation.

## 3. Results

The results of analyses showed that there were no significant differences between the age, sex and duration of phacoemulsification (Table 1).

**Table 1.** Comparing the Age, Sex andPhaco Time of Patients Undergoing Phacoemulsification

Variable	Remifentanil	Propofol	P value
**Mean age, years± SD**	69.94±12.5	70.60±12.4	0.87
**Sex**			0.46
Male	24	23	
Female	26	27	
**phacotime(seconds)**	67.76±19.22	65.16±21.43	0.52

There were no significant differences between the systolic and diastolic blood pressure of the patients before the operation. However, there was a significant difference between the systolic and diastolic blood pressure of the patients in the propofol group while such differences did not occur in the remifentanil group (Table 2).

**Table 2.** Comparison of Hemodynamic Changes in Patients Undergoing Phacoemulsification inRemifentanil and Propofol Condition

Group	Before start of operation	After start of operation	P value
**Remifentanil**			
**SBP^a^(mm/hg)**	135.50 ± 21.20	132.10 ± 20.50	0.41
**DBP^a^(mm/hg)**	87.49 ± 14.64	83.27 ± 12.35	0.12
**HR^a^(bpm)**	62.34 ± 16.37	60.47 ± 17.74	0.58
**Propofol**			
**SBP(mm/hg)**	130.45 ± 18.17	122.32 ± 16.19	0.02
**DBP(mm/hg)**	88.63 ± 13.76	83.23 ±11.7	0.03
**HR(bpm)**	65.54 ± 17.55	58.44 ± 14.17	0.02

^a^Abbreviations: SBP, Systolic Blood Pressure; DBP, Diastolic Blood Pressure; HR, Heart Rate

**Figure 1.**
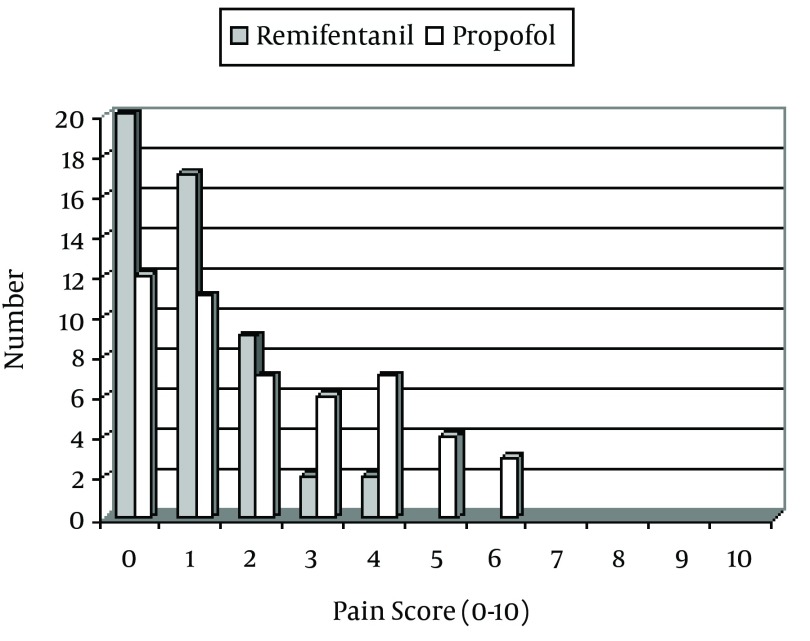
The Frequency Distribution of the Patients According to the Pain Rating Scale During the Operation (VAS)

**Figure 2.**
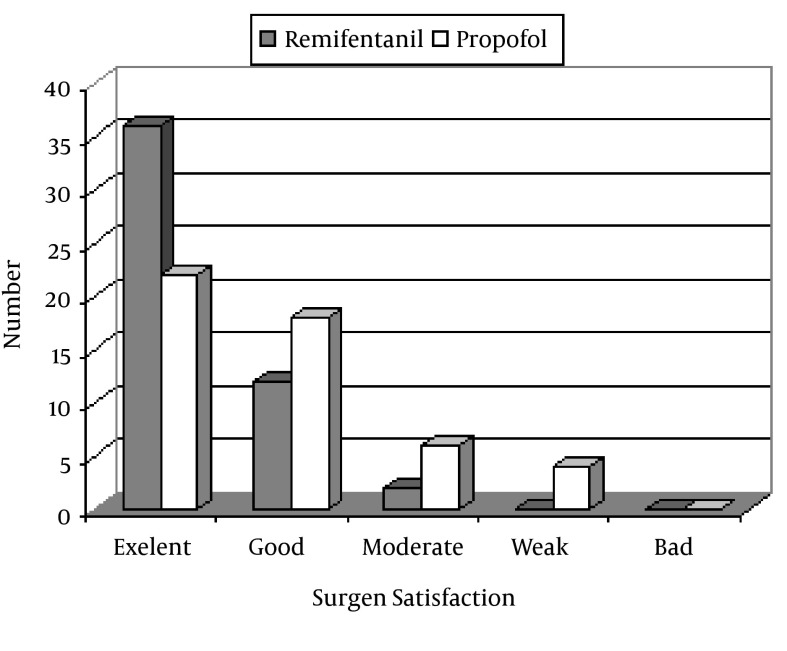
The Frequency Distribution of the Patients According to Satisfaction of Surgeons With Regard to the Patients' Cooperation During the Operation

The mean value of pain for the remifentanil and propofol medications were 1.02 ± 1 and 2.6 ± 1.7, respectively (P = 0.001). The frequencies of pain within 0 to 2 scale was 46 in the remifentanil groups whereas this rate was reported by 30 patients in the propofol condition (P = 0.001). The satisfaction rate of surgeons was higher in remifentanil group (P = 0.01). The frequency distribution of the patients according to the pain rating scale during the operation and the rate of satisfaction of surgeons with respect to the patients' cooperation during the operation are presented in Figures 1 and 2.

Overall, 2 patients in the remifentanil group faced with respiratory depression, however, there was no differences between the two groups (P = 0.24). In addition, no patients in both treatment conditions experienced nausea and vomiting.

## 4. Discussion

The results of this study showed administration of 3 µg/kg/hr of remifentanil compared to 3mg/kg/hr propofol resulted in more pain relief and fewer changes in hemodynamic condition without any more respiratory depression, nausea and vomiting. Researches that assessed the effectiveness of these medicines in patient with topical anesthesia are scarce and the majorities of these in cataract surgery are in the patients with retrobulbar or prebulbar anesthesia or in non-eye surgeries. In this study, the hemodynamic changes in the propofol group was more than the remifentanil group, systolic and diastolic blood pressure was significantly decreased in the propofol group but this change was not statistically significant in the remifentanil group. Lauwers reported similar findings (11). Also, the results of Mingus reported that propofol resulted in significant hemodynamic changes compared to the remifentanil (12). These changes may have occurred due to the known effect of propofol on hemodynamic of the patients and such effect are not so pronounced on hemodynamic response of patient when narcotics such as remifentanil are administered.

In the present research no significant differences between the frequency of nausea and vomiting was found between the two groups. Mingus, (12) in their study reported that administered dose of 12 µg /kg/hr resulted in 60 percent nausea and 21 percent vomiting. In one study conducted by Servin, (13) it was found that the use 6 µg /kg/hr remifentanil caused 26 percent nausea and 6 percent vomiting. By reducing the dose of remifentanil in our study, the frequency of nausea and vomiting was considerably decreased. The results of this study with regard to the frequency of nausea and vomiting was in agreement with the results of the study reported Akcaboy (14), who used remifentanil (0.5 µg/kg followed by 0.05 µg /kg/min) for anesthetic purpose during colonoscopy that did not report any nausea or vomiting. It is reasonable to assume that there is an association between the dose and frequency of nausea and vomiting when the dose is reduced to 3 µg/kg/hr there is limited frequency of these sing and symptoms.

The result of this study showed that the intensity of pain in the remifentanil group was significantly less the propofol group. Akcaboy reported similar results in study when they compared the effect of remifentanil versus propofol during colonoscopy (14). Other investigators also have reported similar findings (11, 13, 14). Since propofol has no pain releving effect in comparison to remifentanil. Remifentanil belongs to the narcotic groups and exerts its effect through the µ receptors, these finding are explainable. In our study, only 2 cases of respiratory depression were observed and after the dose was reduced, this effect was eliminated. In the propofol treatment group, no respiratory depression was observed. In a study that was conducted by Boezaraat (15), a 0.5 mg /kg dose of propofol was compared with 0.3µg/kg remifentanil before the peribulbar block and no respiratory depression was observed. Lawerz (11), showed that anesthesia by using 6 µg /kg/hr dose of remifentanil compared to 3 mg/kg/hr dose of propofol shows more respiratory depression and with reducing the remifentanil dose to 4.5 µ /kg/hr results in elimination of such condition. Servin (13), also reported similar results for the remifentanil group. It seems like there is a relationship between the dose and the respiratory depression and by reducing the dose, the problem is alleviated or removed. With respect to the surgeon satisfaction, remifentanil group significantly had more cooperation with the surgeon. Akcaboy (14), Xu (16), also reported that patients receiving remifentanil had more cooperation with the surgeon in relation to the propofol group. Such findings are probably due to the pain relieving effect of this medicine. The results of this study indicated that using low dose of remifentanil in comparison to propofol results in less pain more surgeon satisfaction without respiratory depression or considerable nausea and vomiting. Further research, in larger group for the evaluation of other medications such as fentanyl and alfentanyl is recommended.

## References

[1] Katz J, Feldman MA, Bass EB, Lubomski LH, Tielsch JM, Petty BG (2001). Adverse intraoperative medical events and their association with anesthesia management strategies in cataract surgery.. Ophthalmology..

[2] Lundstrom M, Stenevi U, Thorburn W (2001). Age-related utilisation of cataract surgery in Sweden during 1992-1999. A retrospective study of cataract surgery rate in one-year age groups based on the Swedish National Cataract Register.. Acta Ophthalmol Scand..

[3] Crandall AS, Zabriskie NA, Patel BC, Burns TA, Mamalis N, Malmquist-Carter LA (1999). A comparison of patient comfort during cataract surgery with topical anesthesia versus topical anesthesia and intracameral lidocaine.. Ophthalmology..

[4] Sullivan KL, Brown GC, Forman AR, Sergott RC, Flanagan JC (1983). Retrobulbar anesthesia and retinal vascular obstruction.. Ophthalmology..

[5] Patel BC, Clinch TE, Burns TA, Shomaker ST, Jessen R, Crandall AS (1998). Prospective evaluation of topical versus retrobulbar anesthesia: a converting surgeon's experience.. J Cataract Refract Surg..

[6] Tsoumani AT, Asproudis IC, Damigos D (2010). Tetracaine 0.5% eyedrops with or without lidocaine 2% gel in topical anesthesia for cataract surgery.. Clin Ophthalmol..

[7] Habib NE, Mandour NM, Balmer HG (2004). Effect of midazolam on anxiety level and pain perception in cataract surgery with topical anesthesia.. J Cataract Refract Surg..

[8] Aydin ON, Kir E, Ozkan SB, Gursoy F (2002). Patient-controlled analgesia and sedation with fentanyl in phacoemulsification under topical anesthesia.. J Cataract Refract Surg..

[9] Sivaci RG, Ermis S, Ozturk F (2005). Fentanyl reduces cortisol and blood glucose changes during cataract surgery under retrobulbar anaesthesia.. Eur J Anaesthesiol..

[10] Kiefer RT, Weindler J, Ruprecht KW (1998). The endocrine stress response after oral premedication with low-dose midazolam for intraocular surgery in retrobulbar anaesthesia.. Eur J Ophthalmol..

[11] Lauwers MH, Vanlersberghe C, Camu F (1998). Comparison of remifentanil and propofol infusions for sedation during regional anesthesia.. Reg Anesth Pain Med..

[12] Mingus ML, Monk TG, Gold MI, Jenkins W, Roland C (1998). Remifentanil versus propofol as adjuncts to regional anesthesia. Remifentanil 3010 Study Group.. J Clin Anesth..

[13] Servin FS, Raeder JC, Merle JC, Wattwil M, Hanson AL, Lauwers MH (2002). Remifentanil sedation compared with propofol during regional anaesthesia.. Acta Anaesthesiol Scand..

[14] Akcaboy ZN, Akcaboy EY, Albayrak D, Altinoren B, Dikmen B, Gogus N (2006). Can remifentanil be a better choice than propofol for colonoscopy during monitored anesthesia care?. Acta Anaesthesiol Scand..

[15] Boezaart AP, Berry RA, Nell ML, van Dyk AL (2001). A comparison of propofol and remifentanil for sedation and limitation of movement during periretrobulbar block.. J Clin Anesth..

[16] Xu ZY, Wang X, Si YY, Wu JC, Zuo YX, Xue FS (2008). Intravenous remifentanil and propofol for gastroscopy.. J Clin Anesth..

